# Investigating the blood-host plasticity and dispersal of *Anopheles coluzzii* using a novel field-based methodology

**DOI:** 10.1186/s13071-019-3401-3

**Published:** 2019-03-25

**Authors:** James Orsborne, Luis Furuya-Kanamori, Claire L. Jeffries, Mojca Kristan, Abdul Rahim Mohammed, Yaw A. Afrane, Kathleen O’Reilly, Eduardo Massad, Chris Drakeley, Thomas Walker, Laith Yakob

**Affiliations:** 1 Department of Disease Control, London School of Hygiene & Tropical Medicine, London, UK; 2 Department of Population Medicine, College of Medicine, Qatar University, Doha, Qatar; 3 Research School of Population Health, College of Health and Medicine, Australian National University, Canberra, Australia; 4 Department of Medical Microbiology, College of Health Sciences, University of Ghana, Korle Bu, Accra, Ghana; 5 School of Applied Mathematics, Fundacao Getulio Vargas, Rio de Janeiro, Brazil; 6 Department of Immunology & Infection, London School of Hygiene & Tropical Medicine, London, UK

**Keywords:** Blood-meal analysis, Host preference, Mosquito, Biting preference, Blood index

## Abstract

**Background:**

The biting behaviour and dispersal of insect vectors in the field underlies the transmission of many diseases. Here, a novel collection methodology coupled with the molecular analysis of blood-meal sources and digestion rates is introduced with the aim of aiding the understanding of two critical and relatively understudied mosquito behaviours: plasticity in blood-host choice and vector dispersal.

**Results:**

A collection strategy utilising a transect of mosquito traps placed at 50 m intervals allowed the collection of blood-fed *Anopheles coluzzii* from a malaria-endemic village of southern Ghana where human host availability ranged from zero (a cattle pen), increasing until humans were the dominant host choice (the middle of the village). Blood-meal analysis using PCR showed statistically significant variation in blood-meal origins for mosquitoes collected across the 250 m transect: with decreasing trend in Bovine Blood Index (OR = 0.60 95% CI: 0.49–0.73, *P* < 0.01) and correspondingly, an increasing trend in Human Blood Index (OR = 1.50 95% CI: 1.05–2.16, *P* = 0.028) as the transect approached the village. Using qPCR, the host DNA remaining in the blood meal was quantified for field-caught mosquitoes and calibrated according to timed blood digestion in colony mosquitoes. Time since blood meal was consumed and the corresponding distance the vector was caught from its blood-host allowed the estimation of *An. coluzzii* dispersal rates. Within 7 hours of feeding, mosquitoes typically remained within 50 m of their blood-host but at 60 hours they had dispersed up to 250 m.

**Conclusions:**

Using this methodology the remarkably small spatial scale at which *An. coluzzii* blood-host choice can change was demonstrated. In addition, conducting qPCR on host blood from field-caught mosquitoes and calibrating with timed experiments with colonised mosquitoes presents a novel methodology for investigating the dispersal behaviour of vectors. Future adaptations to this novel method to make it broadly applicable to other types of setting are also discussed.

## Background

Many disease vectors have demonstrable preference for a particular type of mammalian host to obtain a blood meal, however it is well documented that even the most anthropophilic of disease vectors will still seek a proportion of blood meals from alternative (non-human) host sources [1–4]. Gillies first researched host choice among malaria vectors by releasing *Anopheles* mosquitoes into an enclosed space and comparing the numbers flying into a room holding a human volunteer with those entering a room with a calf [5]. In the subsequent 50 years, the complexity of host preference and biting behaviour has become well documented [2, 6, 7]. While useful, many host-choice experiments have set-ups that can only inform the intrinsic host preference of a vector, and may or may not be indicative of what host species is bitten in natural field settings [8].

Many extrinsic as well as intrinsic factors play a part in who or what is ultimately bitten by a disease vector in a field setting, and these have been summarised comprehensively [2]. This balance between intrinsic and extrinsic factors could go some way to explaining the large variability found in the reported human blood index (HBI) of major disease vectors [2, 9]. Although it has been recognised for a long time that the same mosquito population will often adjust its biting towards a more locally available host species [7, 9, 10], the extent of this plasticity and the spatial scale at which it acts remains understudied even for the most important disease vectors. This plasticity is an important factor when it comes to implementing control strategies. The introduction of insecticide treated nets (ITNs) and indoor residual spraying (IRS) has seen the biting behaviour of many major malaria vectors shift [2, 11, 12] with increasing reports of these vectors seeking blood-meals from alternative non-human sources [3]. Outdoor and residual malaria transmission supported by secondary or indiscriminate malaria vectors [13] further highlights the importance of understanding host choice so future control strategies can be better targeted.

Also implicit to the spatial scale across which feeding choice changes is the vector’s dispersal ability. For example, if a vector tends not to disperse very far, a reasonable assumption may be that it will be less discerning in its choice of host and therefore be more likely to bite whatever is nearby. However, what is of considerable hindrance to this field’s development is the absence of reliable methods for assessing a disease vector’s dispersal ability. Conducting experimental studies on mosquito dispersal has been particularly challenging with the majority of such experiments involving the mark-release-recapture of mosquitoes. However, the impact of handling mosquitoes combined with the typically low recapture rates (in the order of < 2% for *An. gambiae* [14–18]) has limited what can be learned.

Here, the blood-meal sources were identified for *An. coluzzii* caught in traps situated across a 250 m transect representing a range of alternative blood-host species availabilities (primarily human or cattle) in a malaria endemic village of southern Ghana. By using this collection methodology coupled with molecular blood-meal identification, we aim to investigate the spatial range across which this principle malaria vector can adjust its targeted blood-host species based on local host availability. In addition, by quantifying host DNA isolated from field-caught vectors and calibrating this with timed laboratory mosquito feeding experiments, an alternative method is presented for measuring dispersal rates for haematophagous disease vectors. Finally, potential future adaptations to these novel methods are discussed in order to make them broadly applicable to investigating host plasticity and dispersal in other settings.

## Methods

### Study site and mosquito collection

Mosquitoes were collected from the village of Dogo, in the Greater Accra region of Ghana (05°52.418N, 00°33.607E). The village is in the south-eastern coast of Ghana, with the Gulf of Guinea to the south and the Volta River to the east. The average rainfall is approximately 927 mm per year with the main rainy season from April to June and a shorter second season in October. Temperatures range between 23–33 °C. The area is costal savannah with sandy soil, short savannah grass with some small/medium sized trees. The land is used extensively for grazing livestock as well as growing crops for local trade. Housing mostly consisted of concrete structures with concrete/brick walls and flooring. Some traditional mud style houses were also present, more so on the periphery of the village.

**Fig. 1:**
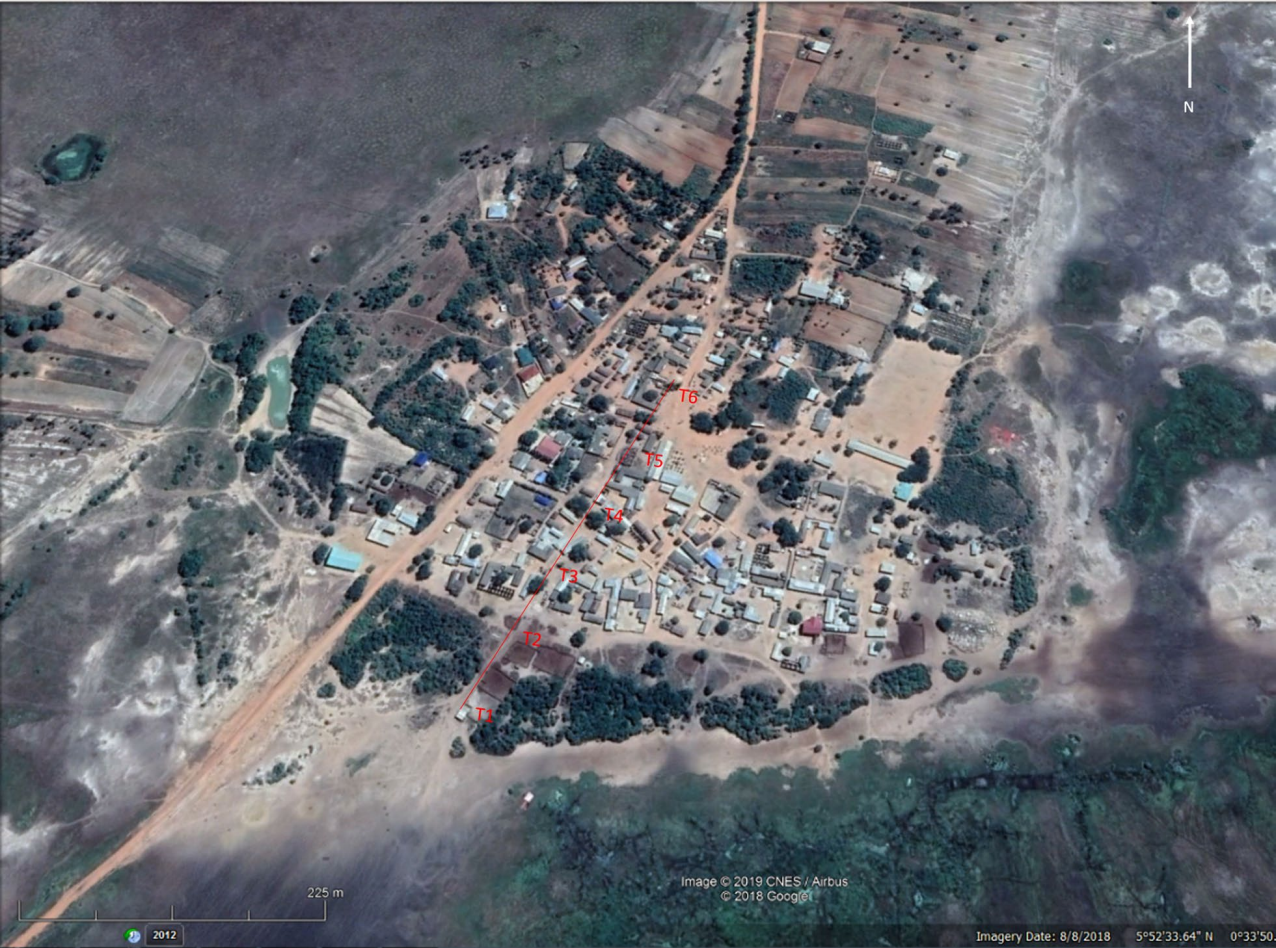
Map of collection site and host species present at each transect point (transect 250 m in total) taken from Google Earth Pro

Mosquitoes were collected across five consecutive nights in June 2017. The trapping setup consisted of CDC resting traps placed outdoors at 50 m intervals forming a 250 m transect comprising of six trapping points (denoted T1–T6). This transect was set beginning at an area of zero human population density (T1, outside of the village by cattle resting and overnight holding pens) and extending towards a human population in 50 m intervals ending at an area of high human density (see Table 1 for description and Fig. 1 for map of collection site). Mosquitoes were collected overnight from 18:00 to 06:00 h.

**Table 1:** Description of areas around transects where mosquitoes were collected including number and type of host present

Transect	Description	Approximate no. of hosts
1	Evening holding pen for cattle for the village (used from 18:00 h to 06:00 h), one small uninhabited house next to the pen was used to hold tools and supplies for cattle farmers	Cows (*n* = 150)
2	End of cattle pen (as described above), small pig holding and empty cattle shed. Edge of village is approximately 30 m away with empty newly built houses; first house with inhabitants (T3) 50 m away	Cows (*n* = 150); pigs (*n* = 5)
3	First cluster of 4 small households on periphery of village *c.*50 m from cattle pen. A small holding of chickens and goats as well as pet dogs which roam the area freely	Humans (*n* = approx. > 20); chickens (*n* = 7); dogs (*n* = 3); goats (*n* = 4)
4	Complex of 5 houses, 3 guinea fowl and 2 cats present, guinea fowl nested in nearby outbuilding, cats roamed freely	Humans (*n* = approx. > 30); guinea fowl (*n* = 3); cats (*n* = 2)
5	Complex of 8 houses, no fixed animal housing	Humans (*n* = approx. > 45)
6	Dogo village, and the largest density of households; one small chicken coop but no other animal holdings, no dogs or cats seen	Humans (*n* = approx. > 85); chickens (*n* = 3)

Mosquitoes were removed from traps at 06:00 h each morning and immediately killed using chloroform to stop any active blood-meal digestion. Mosquitoes where then sorted with all blood-fed females being processed first. All visually blood-fed and gravid *Anopheles* mosquitoes were processed individually with transect location and night collected being recorded. Abdomens of blood-fed mosquitoes were removed with sterile forceps and pressed onto FTA®Classic cards (Whatman, GE Healthcare, New Jersey, USA) to preserve the blood meal for molecular analysis. Excess blood-fed mosquitoes were preserved in RNA later (Thermo Fisher Scientific Life Technologies, Massachusetts, USA) in a 96-well plate where necessary.

### DNA extraction

Mosquito abdomens were extracted individually. Samples were homogenised using a Qiagen TissueLyser II (Qiagen, Manchester, UK) with a 5 mm stainless steel bead (Qiagen) placed in each sample tube in a 96-well plate format. Once homogenised, DNA was then extracted using the Qiagen DNeasy 96 kits (Qiagen) following manufacturer’s protocol. Blood meals preserved on FTA cards were punched out using a sterile steel 4 mm radius punch. Resulting punches were incubated in ATL buffer and Proteinase K for 6 h before DNA extraction was performed following manufacturer’s protocol. Extracted DNA was stored at -20 °C until analysed.

### Mosquito species identification

Mosquito species identification was initiated using a real-time multiplex PCR assay targeting the rRNA gene [12]. Standard forward and reverse primers were used in conjunction with two species-specific Taqman probes. The reaction conditions were as follows: a 12.5 µl reaction containing 1 µl of genomic DNA. 6.25 µl of Quantinova (Qiagen) probe master mix, 800 nM of forward and reverse primers (Thermo Fischer Scientific, East Grinstead, UK), 200 nM of *An. arabiensis* probe (Sigma-Aldrich, Gillingham, UK) and 80 nM of *An. gambiae* probe (Applied Biosystems, UK). Samples were run on a Stratagene MX3005P (Agilent Technologies, Santa Clara, USA) using cycling conditions of 10 min at 95 °C, followed by 40 cycles of 95 °C for 25 s and 66 °C for 60 s. The increases in fluorescence were monitored in real time by acquiring at the end of each cycle.

To differentiate between *An. coluzzii* and *An. gambiae* within the *An. gambiae* species complex, a single end-point PCR was performed. This PCR targets the SINE200 retrotransposon and utilising an insertion in this area allows the two species to be distinguished following gel visualisation [13]. *Anopheles coluzzii* produces a band at 479 bp with *An. gambiae* producing a band at 249 bp. Reaction was as follows: a 25 µl reaction containing 0.5 mM of forward (5′-TCG CCT TAG ACC TTG CGT TA-3′) and reverse (5′-CGC TTC AAG AAT TCG AGA TAC-3′) primers, 12.5 µl of Hot start Taq polymerase (New England Biolabs, Ipswich, UK), 9.5 µl of nuclease-free water and 2 µl of template DNA. Cycling conditions were as follows: 10 min at 94 °C followed by 35 cycles of 94 °C for 30 s, 54 °C for 30 s, 72 °C for 60 s, and a final elongation step of 72 °C for 10 min.

PCR products were visualised on a 2% agarose gel using an Egel E-Gel iBase Power System and E-Gel Safe Imager Real-Time Transilluminator (Invitrogen, East Grinstead, UK). The assay was performed on 10% of all samples identified as *An. gambiae* from the first assay with corresponding controls. Samples producing unknown or inconclusive results were sequenced (ITS2 Sanger sequencing) using primers originally developed by Beebe & Saul [19] and sequences were used to perform nucleotide BLAST (NCBI) database queries. PCR reactions were performed on a T100 Thermal Cycler (Bio-Rad Laboratories, Watford, UK) and amplified gene fragments were visualized by electrophoresis on a 2% agarose gel using an E-gel E-Gel iBase Power System and E-Gel Safe Imager Real-Time Transilluminator (Invitrogen).

### Blood-meal identification

Samples were initially screened using bovine and human specific primers developed by Gunathilaka et al. [20]. These primers were selected based on the abundance of available host species in the area. The reaction conditions consisted of a 10 µl reaction including 0.5 M of forward and reverse primers (Integrated DNA Technologies, Leuven, Belgium), 5 µl of SYBR green master mix (Roche, Welwyn Garden City, UK), 2 µl of nuclease-free water (Roche) and 2 µl of template DNA. PCR was run on a LightCycler 96 real-time PCR machine (Roche) under the following cycling conditions: pre-incubation of 95 °C for 600 s, 40 cycles of 95 °C for 10 s, 62 °C for 10 s and 72 °C for 30 s followed by a melting analysis.

Human-positive blood meals (including any potentially mixed feeds) from the above assay were confirmed using the Promega Plexor^®^ HY Human DNA forensic detection kit (Promega, Southampton, UK). Assay was performed following manufacturer’s protocol using a Stratagene MX3005P (Agilent Technologies, Santa Clara, USA) real-time PCR machine.

### Laboratory assessment of blood-meal DNA degradation rate

Approximately 500 female *An. coluzzii* mosquitoes (N’gousso strain originally collected from Yaounde, Cameroon) were placed into an insect cage (Bugdorm, Watkins and Doncaster, UK) and, using a Hemotek, fed for 15 min on bovine blood collected from a UK based abattoir (First Line UK (Ltd), UK). Mosquitoes were reared at the London School of Hygiene & Tropical Medicine under standardized conditions in an incubator (27 °C and 70% humidity with a 12:12 light/dark photocycle) and given access to 10% sugar solution. Female mosquitoes were individually collected and checked for feeding status. Only overtly fully fed mosquitoes were selected for the experiment. Fully-fed females were separated into paper cups covered with netting; each cup contained a maximum of 30 female mosquitoes. Every 6 hours a single cup was removed and placed in a -80 °C freezer to kill the mosquitoes and stop blood-meal digestion. This was repeated until the mosquitoes had completely digested the blood-meal or were visually gravid. DNA was extracted using the above protocol from seven whole bodies for each time point. A 1:10 serial dilution of all time = 0 samples was used to generate a standard curve with dilutions being made down to 1 × 10^-7^. The standard curve was used to assess assay sensitivity (limit of detection) with the resulting Ct values from each time point being used to estimate the time post-blood-feed for the field-caught mosquitoes. DNA from the blood meals from the field-caught mosquitoes was quantified using the same protocol. As larger female mosquitoes typically obtain a larger blood meal when feeding [21], we normalised for mosquito body size to account for the possibility that the different quantity of bovine DNA across the transect was due to mosquito size rather than time post blood-meal. Ct values for bovine DNA were normalised against the Ct values for the corresponding host mosquito ribosomal DNA (rDNA) gene used for species identification, producing a ratio of bovine (*Bos taurus* mtDNA)-to-vector DNA (*An. coluzzii* rDNA). In this way, the quantity of bovine DNA measured for the timed experiments with colonised mosquitoes was used to estimate the time since last blood meal of the mosquitoes caught at the different transect points. In conjunction with the known distances between the hosts and the transect points, this estimated time since last blood meal informed the dispersal rate of the vectors.

### Statistical analysis

All statistical analysis was performed using STATA and PRISM. Trends in blood indices across the transect were tested for the field-caught mosquitoes using a generalised linear model (glm) with a binomial function. Odds ratios were calculated for proportion of bovine or human fed mosquitoes across each collection night as a total of *An. coluzzii* collected and *P*-values (*P* < 0.05) were used to interpret any significant trends. Linear regression was performed to investigate the correlation between bovine Ct value and time post-feed recorded in the experiments with colony insects.

## Results

A total of 318 blood-fed *Anopheles* mosquitoes were collected over a five-night period. Of these, 307 were identified as part of the *An. gambiae* species complex: 306 were identified as *An. coluzzii* using a combination of species-specific PCRs and Sanger sequencing of a fragment of the ITS2 region. The remaining insect was identified by ITS2 Sanger sequencing as *An. melas* and was excluded from the analysis (Table 2).

**Table 2:** Total number of blood-fed mosquitoes caught by species and transect point

	T1	T2	T3	T4	T5	T6	Total
*An. coluzzii*	17	153	72	26	22	16	306
*An. melas*	0	1	0	0	0	0	1
Other species	0	9	2	0	0	0	11
Total	17	163	74	26	22	16	318

**Fig. 2:**
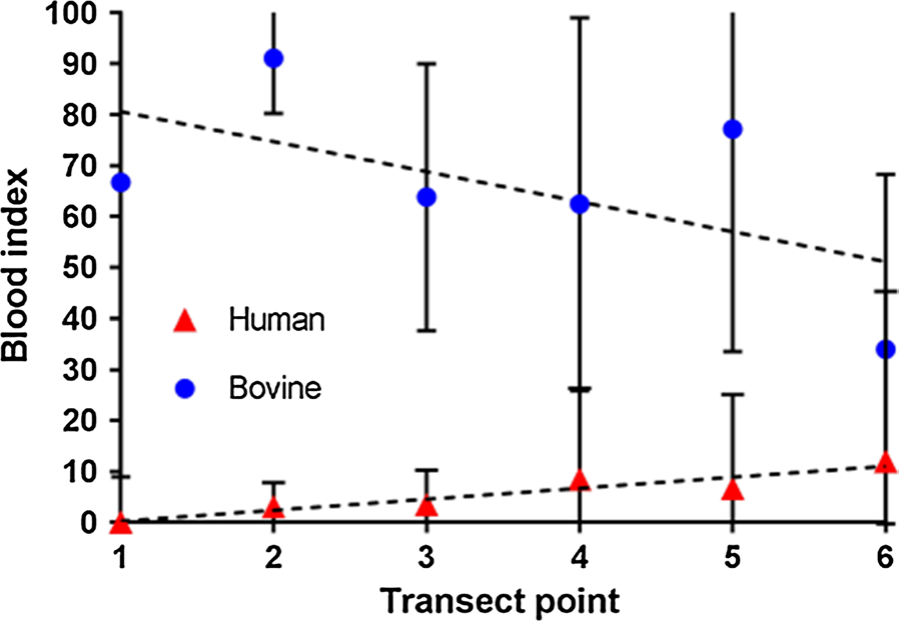
The human blood index (triangles) and bovine blood index (circles) for each transect point (T1-T6), along with 95% confidence intervals, for all blood-fed *An. coluzzii* mosquitoes collected

The dominant mosquito blood meal was of bovine origin with 73.5% of all meals being sourced from these hosts. Four (1.3%) individual mosquitoes were found to have solely fed on humans with an additional ten (3.3%) having a mixed feed of both bovine and human blood (Table 3). Figure 2 shows how the bovine blood index (BBI) varied significantly across the transect, indicating a decreasing trend with increasing distance from the cattle shed (OR = 0.60, 95% CI: 0.49–0.73, *P* < 0.01). The opposite trend was observed for human blood meals with the HBI increasing significantly towards the village (OR = 1.50, 95% CI: 1.05–2.16, *P* = 0.028).

**Table 3:** Total number of *An. coluzzii* mosquitoes collected by blood-meal source and transect point

Host source	T1	T2	T3	T4	T5	T6	Total	%
Bovine fed	15	138	39	12	18	5	227	74.18
Confirmed human feds	0	1	0	1	1	1	4	1.31
Mixed human/bovine	0	5	2	1	0	2	10	3.27
Unknown	2	9	31	12	3	8	65	21.24
Total caught	17	153	72	26	22	16	306	100

Focusing on mosquitoes that had fed on cattle (*n* = 227), it was observed that the quantity of blood-host DNA extracted from mosquitoes varied across the transect with the average PCR cycle threshold (Ct) values for bovine blood detection being 20.72 (95% CI: 18.98–22.45) for mosquitoes caught by the cattle pen and 30.15 (23.14–37.16) for mosquitoes caught 250 m away (*P* < 0.01). As detection of rDNA is a proxy for total mosquito DNA extracted and therefore body size, we compared the ratios of bovine-to-vector DNA (*An. coluzzii* rDNA) across the transect to ensure different quantities of bovine DNA detected at different distances from the hosts was not due to mosquito size but rather time post-blood-meal. The correlation between Ct ratio and distance from cattle was retained (*t*_(225)_ = -2.18, *P* = 0.03).

**Fig. 3:**
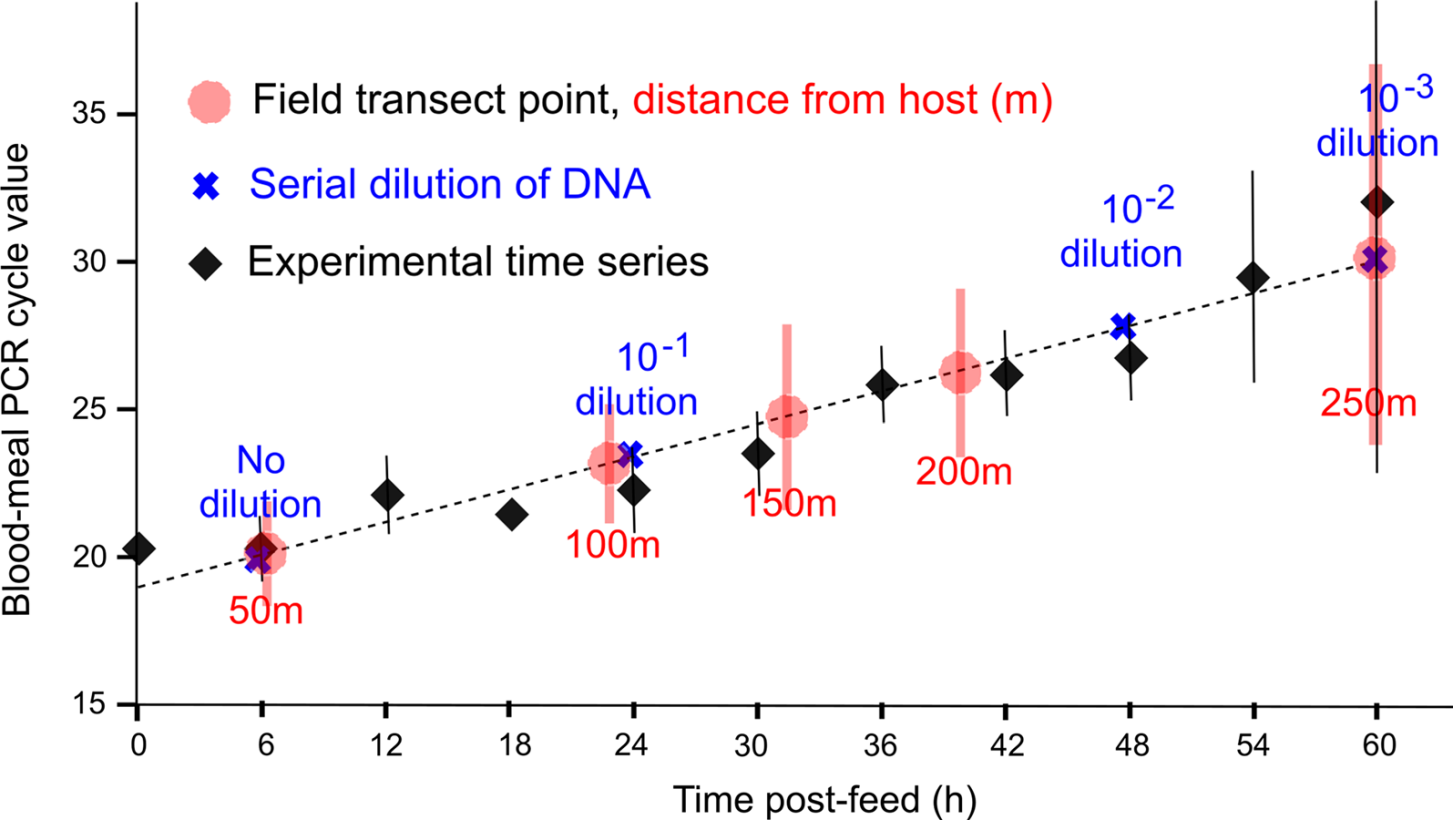
Effect of time post blood-meal on mean bovine Ct values produced from qPCR. Shown are the means (bars indicate 95% CIs) of experimental time series (black), the serial dilution Ct values to assess assay sensitivity (blue), the mean (and 95% CI) Ct values of each transect point (red) and regression line used to predict time post feeding (dashed black line). Note that ‘time post-feed’ is from direct observation for colony mosquito blood-meal digestions (black) but is then extrapolated to the estimated time post-feed for field-caught mosquitoes

The experimental time series was performed with a laboratory colony of *An. coluzzii* and producing mean Ct values for known time points post-blood-feeding. The time series showed Ct values increased with time post-feed (*P* < 0.01, see Fig. 3) with no bovine DNA detected after the 60-hour time point. Regression analysis showed a positive correlation between bovine Ct value and time post-feed in the experimental time series (*R*^2^ = 0.92, slope = 0.183; see Fig. 3). Calibrating the blood-meals of field-caught mosquitoes using the timed experiment with our mosquito colony, the dispersal rate of *An*. *coluzzii* could then be extrapolated: within 7 hours of feeding, mosquitoes typically remained within 50 m of their blood-host but at 60 hours had dispersed up to 250 m (Fig. 3).

## Discussion

Evidence for the influence of local host availability on blood-host selection was demonstrated through analysis of the blood meals of *An*. *coluzzii* caught from the field using a novel sampling strategy. Previous investigations of HBI in field settings have demonstrated its large variability across and within species [2]. The aim of the present study was to investigate, for the same mosquito population, what level of variability can be expected, and, to determine the spatial scale that this choice can vary.

Here, a relatively low-cost (the chief expense being the PCR for blood-host species identification) and simple experimental setup for investigating host choice in the field is described. It was demonstrated that local host availability plays a crucial role in the host choice of a major malaria vector. Moreover, the remarkably small spatial scale (~250 m) at which this behaviour can be significantly impacted is demonstrated for the first time.

Results could have significant implications for vector control. For example, field studies involving endectocidal applications on livestock have shown encouraging results in terms of long-lasting mosquitocidal effects [22, 23]. However, previously this strategy has only been considered for targeting malaria vector species traditionally viewed as zoophilic (e.g. *An*. *arabiensis*). Recently, this assumption has been challenged by the demonstration that even the most anthropophagic populations of vectors readily bite non-human hosts, and that the methods for assessing host choice exclusively from mosquitoes caught in human habitation may suffer from systematic bias [9]. Therefore, one way by which the current study adds to the discussion of optimal vector control strategy is through the provision of a simple method for assessing the degree to which anthropophagy varies for a given mosquito population. This can then be used to inform strategies for improved targeting of different control methods such as endectocides. Coupled entomological-epidemiological modelling frameworks already exist for using these data to inform projections of this novel vector control [24], including its use as part of an integrated vector management programme [25].

Linking the quantity of host-blood DNA isolated from mosquitoes caught at known distances from the specific host species with timed blood-meal digestion assays conducted on colonised mosquitoes presents a novel method for informing dispersal rates of mosquito populations. Dispersal is recognised to underlie mosquito population structure [17] as well as human exposure to transmission [26] and our ability to control transmission [27]. Yet, knowledge of this critical aspect of behaviour has been hampered by our inability to produce reliable estimates of vector dispersal in the field. To our knowledge, this study provides the first estimates using a non-intrusive and easily repeatable method for measuring malaria vector dispersal that informs the mosquito’s dispersal rate across its gonotrophic cycle (approximately 2.5 days). However, it is important to address some of the present study’s limitations and to identify some areas of future development of this approach.

First, in this study the numbers of mosquitoes captured nearby humans was low compared to those caught adjacent to cattle. That said, only 5 nights of mosquito captures were needed in order for a statistically significant trend to be identified for host choice across the transect. In the future, increasing the duration of the experiment would improve its ability to inform the likely shape of dispersal (e.g. leptokurtic *versus* Gaussian), something that could not be achieved with the present study.

Secondly, in order to estimate distances from blood-hosts these hosts must remain spatially confined. While this was possible in the present study because cattle were confined to their holding pen, this may require experimental adaptations for other types of environment. It must be made clear that this experiment in this particular field site was not intended to inform *An. coluzzii* dispersal rates everywhere that this vector can be found. Rather, the aim of this study was to present a new method for measuring dispersal that can be adapted to other settings to inform local mosquito behaviour. For example, tethering an animal species not otherwise found in the vicinity of a field site, followed by identifying its DNA from blood-fed mosquitoes caught nearby is one such setup that requires future investigation.

Thirdly, blood-meal digestion levels of field-caught mosquitoes were calibrated with colonised mosquitoes. Here multiple differences can occur: colony fed mosquitoes are reared at controlled densities, temperature and humidity and are able to take a full blood meal without encountering any defensive behaviour from hosts. These are of stark difference to what blood-fed mosquitoes may encounter in the field. A realistic temperature/humidity regimen that better emulates natural diurnal patterns has been shown to significantly impact various aspects of mosquito metabolism [23]; and, artificially controlling larval density can produce mosquitoes of similar size and fitness, something which may not be comparable to the field. Future experiments to ascertain the influence that these factors may have on blood-meal digestion would constitute an important next step.

## Conclusions

Results presented in this study provide new insight into fundamental aspects of malaria vectors with important implications for malaria control strategy. Additionally, the novel experimental design presented offers a new methodology in measuring dispersal that with further development could be broadly applicable to other field-caught blood-feeding disease vectors.

## Abbreviations

HBI: human blood index
BBI: bovine blood index
Ct: cycle threshold
OR: odds ratio
CDC: centers for disease control and prevention
